# Investigating the role of the brain-derived neurotrophic factor Val66Met polymorphism in repetitive mild traumatic brain injury outcomes in rats

**DOI:** 10.1186/s12993-025-00270-5

**Published:** 2025-03-05

**Authors:** Lauren P. Giesler, William T. O’Brien, Jesse Bain, Gershon Spitz, Emily J. Jaehne, Maarten van den Buuse, Sandy R. Shultz, Richelle Mychasiuk, Stuart J. McDonald

**Affiliations:** 1 Department of Neuroscience, Monash University, Melbourne, Australia; 2 Monash-Epworth Rehabilitation Research Centre, Monash University, Melbourne, Australia; 3 Department of Psychology Counselling and Therapy, School of Psychology and Public Health, La Trobe University, Melbourne, Australia; 4 Health Sciences, Vancouver Island University, Nanaimo, BC Canada; 5 Department of Neurology, The Alfred Hospital, Melbourne, Australia; 6 Department of Neuroscience, School of Translational Medicine, Monash University, 99 Commercial Road, Melbourne, VIC 3004 Australia

**Keywords:** Concussion, Val66Met, Biomarker, Microglia, Neurofilament light, Behavior

## Abstract

**Background:**

Mild traumatic brain injury (mTBI) poses a significant public health concern, particularly regarding repetitive injury, with outcomes ranging from acute neurobehavioral deficits to long-term impairments. While demographic factors like age and sex influence outcomes, the understanding of genetic contributions, particularly the role of the brain-derived neurotrophic factor (BDNF) Val66Met polymorphism, remains limited. This study aimed to characterize acute effects of repetitive mTBI (rmTBI) in rats with the Val68Met SNP, the rodent equivalent of the human Val66Met, focusing on behavioral, fluid biomarker, and histological changes.

**Methods:**

Using a closed-head injury model, rats underwent five mTBIs over consecutive days. Behavioral assessments included sensorimotor function, anxiety-like behavior, spatial learning and memory, and nociceptive response. Plasma neurofilament light (NfL) levels served as a biomarker of axonal injury and immunohistochemistry evaluated microglial activation.

**Results:**

Sensorimotor deficits and increased anxiety-like behavior were found in rats with rmTBI, but these changes were not affected by sex or genotype. Plasma NfL levels were higher in rmTBI compared with sham rats, with levels greater in female rmTBI when compared with male rmTBI rats. Microglial activation was observed in the hypothalamus of injured rats, but was not influenced by genotype or sex.

**Conclusions:**

While the Val68Met SNP did not significantly influence acute responses to rmTBI in this study, further investigation into alternative functional and pathophysiological outcomes, as well as long-term effects, is required.

**Supplementary Information:**

The online version contains supplementary material available at 10.1186/s12993-025-00270-5.

## Background

Mild traumatic brain injury (mTBI) is a serious public health concern, often resulting in a variety of neurobehavioral deficits [1, 2]. Clinically, mTBI is characterized by a Glasgow Coma Scale score of 13 to 15, impaired mental state post-injury, a brief loss of consciousness (if any), and < 24 h of post-traumatic amnesia [3]. While symptoms may be transient, mTBI is associated with an increased risk of developing long-term neurological conditions, particularly when injuries are repetitive [4, 5]. The outcomes of mTBI are incredibly heterogeneous, with individuals experiencing a wide spectrum of symptoms and recovery trajectories [6]. Several demographic factors such as age and sex are known to influence outcomes after repetitive mild traumatic brain injury (rmTBI) [7, 8]. However, our comprehension of how genetics contributes to and could be used to prognosticate recovery from rmTBI is at an early stage of development.

Brain-derived neurotrophic factor (BDNF) may play an important role in the outcomes of rmTBI given its essential involvement in neuronal growth, survival, and differentiation [9, 10]. The Val66Met single nucleotide polymorphism (SNP) causes a valine (Val) to methionine (Met) amino acid substitution at codon 66 of the BDNF pro-domain, which ultimately results in diminished activity-dependent secretion of BDNF in individuals carrying the Met variant [11, 12]. Studies have shown that the Val66Met influences the sorting and cleavage of proBDNF, which may result in higher concentrations of both proBDNF and mature BDNF in neuronal cell bodies rather than in dendrites [13, 14]. This alteration of the secretory pathway hinders the release of mature BDNF from dense core vesicles at the synaptic terminals [15]. This SNP has been associated with a plethora of neurobehavioral impairments, including deficits in cognition [16, 17], increased anxiety [18–20], and increased depressive symptoms [21, 22]. The Val66Met polymorphism has also been associated with impairments in both motor function [23, 24] and nociceptive response [25].

The activation of microglia is one of the most prominent inflammatory responses to brain injury. Following acute brain injury, microglia can change phenotype and migrate towards the site of damage [26]. Rodent studies investigating mTBI have shown significant increases in ionized calcium-binding adaptor molecule 1 (Iba-1) or activated microglia following TBI and rmTBI at acute and chronic timepoints [27–29]. Recent findings highlight that BDNF may influence microglial activation dynamics in TBI by modulating TrkB signaling [30]. Specifically, reduced availability of BDNF or altered signaling resulting from the Val66Met genotype, may impair resolution of microglial activation, thus prolonging neuroinflammatory responses [30]. Stroke research in rodents has shown that in the inflammatory process, BDNF may promote microglial proliferation and phagocytic activity [31]. It is proposed that the promotion of proliferation and activity creates a feedback loop wherein increased proliferation and activity of microglia lead to elevated levels of both phagocytic and activated microglia, which in turn release BDNF [31, 32]. An in vitro model revealed that BDNF treatment following lipopolysaccharide-induced inflammation reversed the release of interleukin-6 and tumor necrosis factor alpha in cortical primary microglia, indicating that BDNF might play a role in the anti-inflammatory response triggered by microglia [33]. Moreover, a study focusing on the inflammatory response following rmTBI in Val68Met mice demonstrated a significant increase in activated Iba-1 cells in the hippocampus and cortex of Val/Met mice compared with wildtype Val/Val mice [27].

The relationship between neurofilament light (NfL) and BDNF, while underexplored, may be significant in the context of rmTBI. With NfL as a common marker of axonal damage after injury [34], and BDNF playing a role to support axonal integrity and repair [35], it is hypothesised that impaired BDNF signaling resulting from the Val66Met SNP may exacerbate axonal vulnerability, leading to increased NfL levels following injury.

Despite these findings, there is limited research into the role that the Val66Met polymorphism has on behavioral and molecular outcomes in the context of rmTBI. Owing to this SNP’s prevalence, occurring in roughly one fifth of the global population [36], it is important to understand how its underlying genetic mechanisms may influence recovery in Val66Met carriers exposed to rmTBI. Here, we aimed to characterise the acute behavioral and neuropathological effects of rmTBI in rats with the Val68Met (i.e. the rodent equivalent of the Val66Met) SNP. To do so, we implemented an extensive investigation of behavioral outcomes and changes in platelet poor plasma NfL levels and Iba-1 + cells in rats known to carry different variations of the BDNF SNP (i.e. Val/Val, Val/Met, Met/Met) following five mTBIs.

## Methods

### Subjects

In total, 114 Sprague-Dawley (60 Males, 54 Females) rs6265 (Val68Met) rats were obtained from a breeding colony set up at the Alfred Medical Research and Education Precinct (AMREP) Animal Services (Melbourne, Australia) [37–39] (Supplementary Fig. 1). All animals were genotyped (i.e. Val/Val, Val/Met, or Met/Met carriers) at weaning via ear punch by Transnetyx (Cordova, TN, USA). Rats were housed in cages of 3 on a 12-hr light/dark cycle with *ad libitum* access to water and chow. Behavioral testing was conducted during the light phase of the light/dark cycle.

### mTBI

**Fig. 1:**
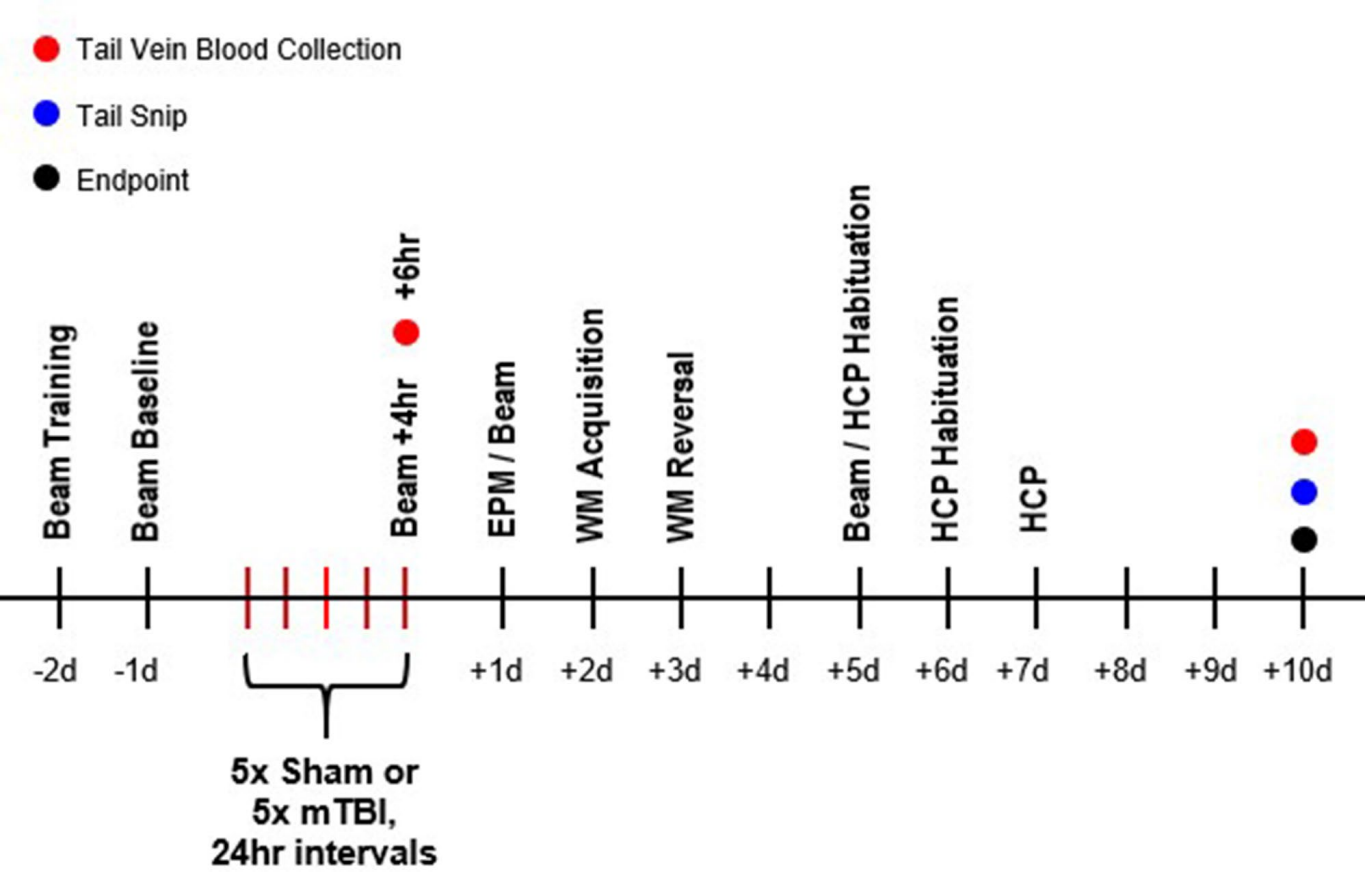
Timeline of behavioral testing. Animals underwent a behavioral battery over 10 days post-injury. Two animals were excluded from battery due to post-injury mortality (1 male Met/Met, 1 female Met/Met)

The awake closed-head injury (ACHI) model was used to induce rmTBI [40]. Rats were pseudo-randomly assigned to either rmTBI (*n* = 56) or sham (*n* = 58) conditions. Two days prior to the initial injury, rats underwent habituation. Rats were restrained using a Decapicone™ bag and briefly placed on a foam platform. One day prior to initial injury, rats underwent a similar habituation, but were fitted with a steel helmet following restraint. On the day of injury, rats were restrained, fitted with a steel helmet, placed on a foam platform, and a controlled cortical impactor device (Leica Biosystems, IL, USA) was then used to drive a 5 mm impact tip into the helmet centred over the left parietal bone. Injuries were performed once daily for 5 days (Fig. 1). Sham procedures were identical to rmTBI procedures, but in absence of impact.

## Behavioral testing

### Beam task

Sensorimotor function was assessed through a series of beam task evaluations conducted at multiple time points: baseline (1 h prior to the first injury), and 4 h, 24 h, and 5d after the final injury. The beam task protocols closely followed the procedures outlined in the work of O’Brien et al. (2021) [40]. The experimental apparatus consisted of a 2 cm wide, 1.5 m long wooden beam, elevated 75 cm above the ground. An intense bright light, serving as an aversive stimulus, was positioned at the starting end of the beam, while a dark box was placed at the opposite end. To prepare the rats for the testing, they underwent two training sessions on the beam, with the first session taking place 48 h before the baseline assessment and the second one 24 h before. In the training sessions, rats initially crossed a 4 cm wide beam. They underwent two trials at distances of 25 cm, then 50 cm, and finally 75 cm. This process was repeated on the 2 cm beam in the same manner. For testing, each rat was positioned on the 2 cm wide beam and required to traverse a distance of 75 cm for a total of five trials. Rats unable to finish a trial within 20s were assigned to this maximum value. Rats that fell from the beam were assigned the maximum time in addition to 2 hindlimb slips. These trials were captured on video for subsequent analysis of hind limb slips and traverse times by a researcher blinded to the experimental conditions.

### Elevated plus maze

The elevated plus maze was used to assess anxiety-like behavior at 24 h post-final injury. The maze comprised of two open arms and two enclosed arms extending from a central platform, elevated 50 cm above the ground. To initiate the test, the rat was placed at the centre of the maze, facing one of the open arms, and allowed to explore the maze freely for 5 min. An overhead camera captured the trial, and the rat’s movements were analyzed using TopScan software. Time spent in both open and closed arms was recorded, with greater time spent in closed arms indicating heightened anxiety-like behavior [39].

### Water maze

Spatial learning and memory were assessed at 48- and 72 h post-final injury using the water maze in a protocol similar to that described by Pham et al. (2021) [41]. A 175 cm diameter circular pool was filled with 28 $\:\pm\:$ 1 °C tap water. The arena was separated into four quadrants with four unique visual stimuli fixed over the edge of each quadrant as cues for spatial orientation. A hidden escape platform was placed 3.5 cm below the surface of the water in one quadrant. Testing involved an acquisition session at 48 h and a reversal session at 72 h post-final injury. Each session consisted of ten trials with a maximum trial time of 60s. At the start of each trial, rats were pseudo-randomly placed in one of the quadrants, facing the wall of the pool. The trial ended when the rat located the hidden platform. An overhead camera captured each trial and TopScan software was used to track the animal. The reversal settings were identical to those used during acquisition, except that the hidden platform was relocated to the opposite quadrant prior to the start of the trials. Latency to find the platform was used as a measure of spatial learning (acquisition) and memory (reversal).

### Hot/cold plate

Hot cold plate testing, as described by Salberg et al. (2021) was used to measure nociceptive response [42]. Rats underwent two days of plate habituation at 5- and 6d post-injury. During habituation, rats were placed on a room-temperature plate enclosed by a transparent cylinder for 2 min to acclimate to the apparatus. Testing was performed at 7d post-injury. During testing, the plate was initially heated to 52 °C, the rat was placed into the apparatus, and immediately removed upon the elicitation of a reaction. The rat was returned to the home cage where it was left undisturbed for 90 min. The testing was then repeated with the plate cooled to 2 °C. Latency to react (i.e. lifting a hind paw or licking a paw) to hot and cold temperature was recorded, with greater latency indicating increased nociceptive thresholds.

### Tissue and blood collection

Six hours after the final injury, rats were briefly anesthetised via isoflurane inhalation and a 23-gauge needle was used to collect whole blood from the lateral tail vein. Blood was collected into BD Microtainer^®^ K2 EDTA tubes and centrifuged at 1100 g for 10 min at 4 °C. Samples then underwent an additional centrifugation at 10,000 g for 10 min at 4 °C to collect platelet poor plasma. Plasma was then stored at -80 °C. At 10d post-injury, rats were euthanised for collection of fixed tissue. Rats were anesthetised and injected with Lethabarb^®^ (160 mg/kg), and following cardiac blood collection, rats were transcardially perfused with phosphate-buffered saline (PBS) followed by 4% paraformaldehyde (PFA). Whole brains were extracted and post-fixed with 4% PFA at 4 °C for 24 h. Brains were then washed 3 times with 1x PBS before being stored in 1x PBS at 4 °C for later histological processing.

### Plasma NfL quantification

A Simoa HD-X Analyzer (Quanterix, Billerica, MA, USA) was used to quantify platelet poor plasma NfL (Simoa NF-light Advantage Kit), following manufacturer’s instructions. All samples were tested in duplicate and measured above the lower limit of detection (0.038 pg/mL). The average coefficient of variation (CV) for duplicate samples was 4.2%. Control samples were run on each plate with an average interplate coefficient of variation of 6%. Due to insufficient quantity of viable samples, 6 h post-injury analysis could not be conducted.

### Immunohistochemistry

Seventy-two (i.e., *n* = 6 per genotype/injury group/sex) rat brains were submitted to the Monash Histology Platform for immunostaining to evaluate microglia. Two coronal sections (20 μm) were collected at bregma − 5.0 for staining. Frozen sections were thawed and postfixed for 5 min in 10% neutral buffered formalin. Antigen retrieval was performed in DAKO PT Link in 1x DAKO Target Retrieval (Low PH) Solution at 98 °C for 15 min. Slides were then washed twice in 1x DAKO EnVision Flex Wash Buffer for 5 min at room temperature (RT), followed by a peroxidase block (Real Peroxidase Blocking Solution, DAKO) for 15 min at RT. Slides were washed again and protein block (Serum Free Protein Block, DAKO) was applied at RT for 30 min, then tipped off of the slides. Sections were incubated overnight in primary antibody (rabbit anti-Iba-1, 1:6000, Wako USA Inc, VA). Slides were washed 3x and secondary antibody (EnVision System-HRP Labelled Polymer Anti-Rabbit, DAKO) was applied for 2 h at RT. Sections underwent 3 × 10 min washes before DAB (Liquid DAB + Substrate Chromogen, DAKO) was applied for 2 min at RT. Slides were again rinsed in dH_2_O. Slides were dehydrated in ethanol, cleared in xylene, and coverslipped with DPX mounting medium.

### Microglial cell count & percentage area

**Fig. 2:**
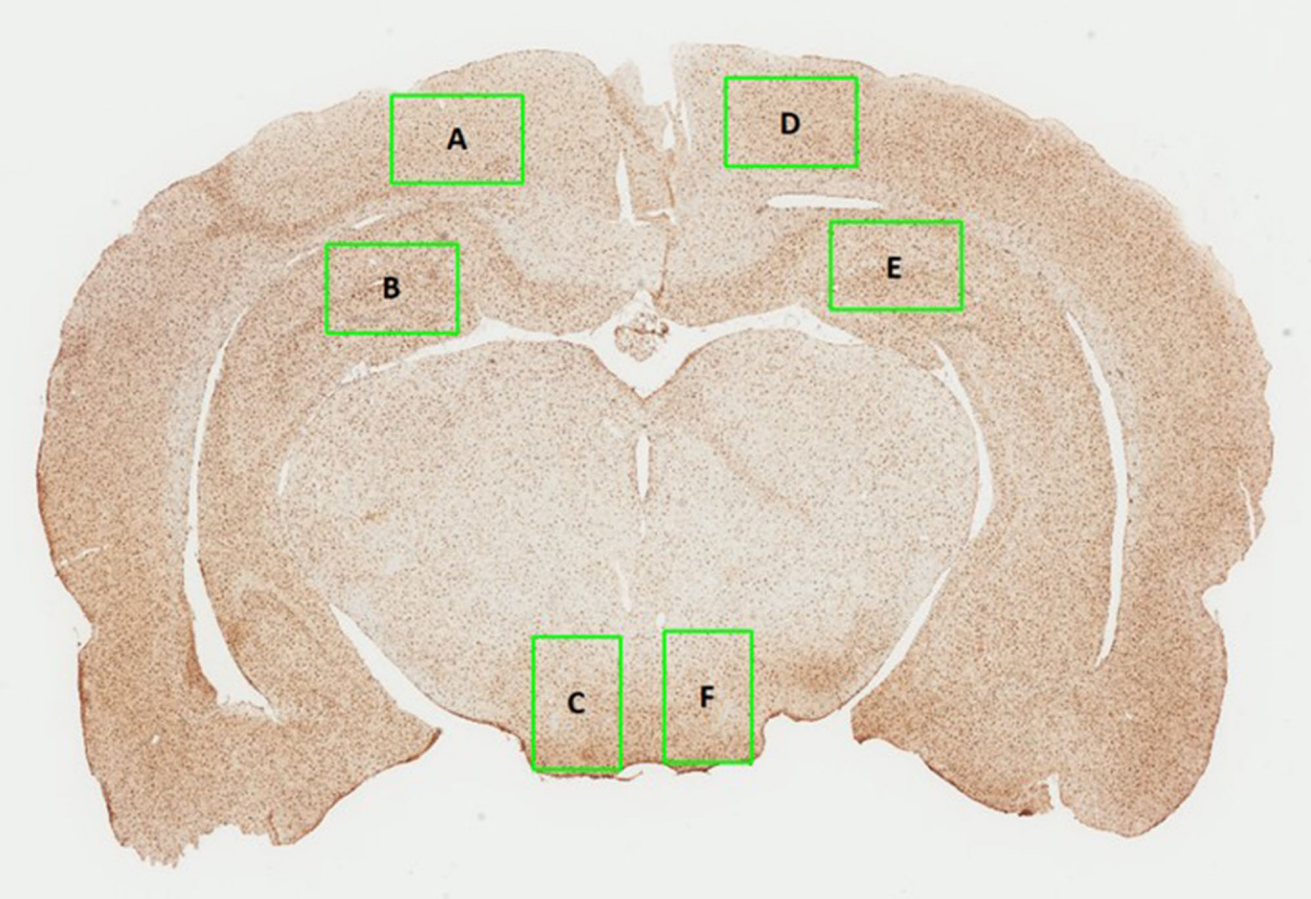
Brain regions analysed for Iba-1 immunohistochemistry. Regions analysed included the (**A**) ipsilateral cortex, (**B**) ipsilateral hippocampus, (**C**) ipsilateral hypothalamus, (**D**) contralateral cortex, (**E**) contralateral hippocampus, and (**F**) contralateral hypothalamus

To quantify microglia, 1 mm x 1.5 mm boxes were positioned over the regions of interest - ipsilateral and contralateral to injury– in the cortex, hippocampus, and hypothalamus (see Fig. 2, using ImageScope (v12.4.6.5003). A researcher blinded to the experimental groups manually counted the number of Iba-1 positive cells in each region of interest for two consecutive brain sections and reported the average cell count (Supplementary Fig. 2). To quantify percentage area of Iba-1 positive cell bodies, images were uploaded to ImageJ (v1.54 g), converted to 8-bit, and a custom threshold was applied to control for inter-batch variations in staining. One or more regions of interest across five brains (1 Val/Met TBI, 3 Met/Met TBI, 1 Val/Val Sham) were unable to be quantified due to poor section quality.

### Statistics

Behavioral outcomes (excluding beam task and water maze), immunohistochemistry outcomes, and NfL biomarker outcomes were analysed with three-way analysis of variance (ANOVA), with a significance level set to < 0.05, using GraphPad Prism (v9.3.1) to investigate the relationship between injury, genotype, and sex. Post-hoc analyses were performed with Sidak’s multiple comparisons test. Plasma NfL data underwent natural logarithm transformation prior to analysis. Four-way repeated measures ANOVAs with timepoint, sex, injury, and genotype as factors were conducted in SPSS 28.0 to examine the beam task and water maze data.

## Results

### Beam task

**Fig. 3:**
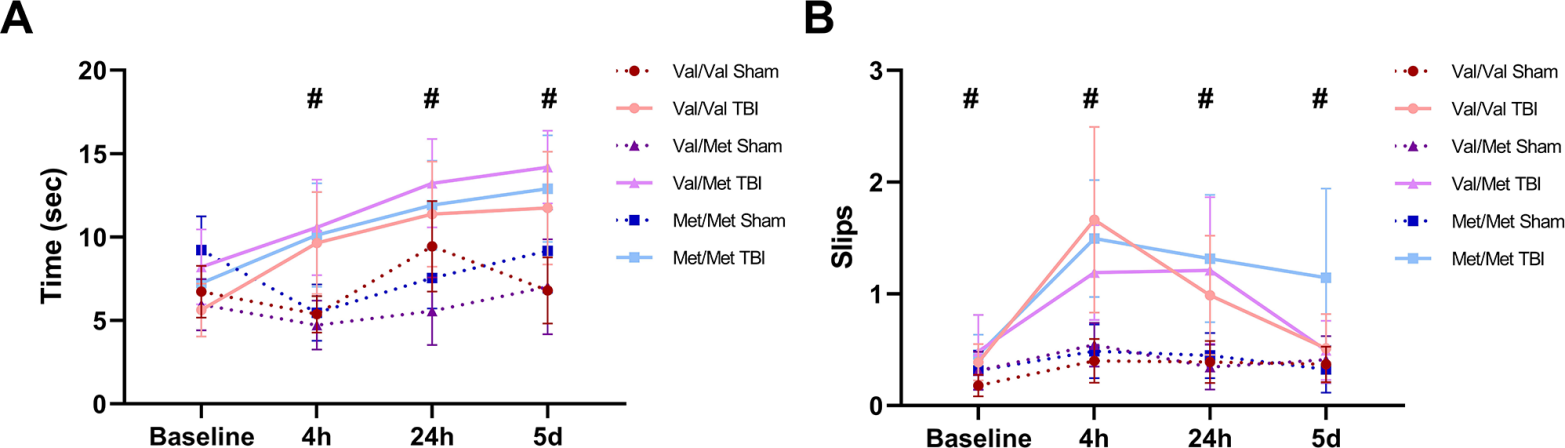
Sensorimotor function measured by the beam task. (**A**) Average time to traverse the beam. There was an injury-time interaction on traverse time (*p* < 0.001). (**B**) Average number of hindlimb slips. There was an injury-time interaction on hindlimb slips (*p* < 0.001). Sham = 58 (Val/Val = 19, Val/Met = 18, Met/Met = 21), rmTBI = 54 (Val/Val = 16, Val/Met = 19, Met/Met = 19). Data presented as mean ± SEM. # indicates a significant injury-time interaction

The average time to traverse the beam and average number of beam slips are shown in Fig. 3A and B, respectively. For the beam traverse time, Machley’s test of sphericity was violated (*p* < 0.001) and lower-bound corrections were used. There was an interaction between injury group and timepoint (F = 14.181, *p* < 0.001), with injured animals taking longer to traverse the beam than shams at 4 h (MD = 4.89, 95%CI: 3.11–6.67, *p* < 0.001), 24 h (MD = 4.66, 95%CI: 2.77–6.54, *p* < 0.001), and 5d (MD = 5.30, 95%CI: 3.22–7.37, *p* < 0.001) post-injury. There was no effect of either genotype or sex, nor any interaction, on beam traverse time. Within the beam slips, Machley’s test of sphericity was not violated and sphericity was assumed. There was an interaction between injury group and timepoint (F = 8.771, *p* < 0.001), with injured animals having more hindlimb slips than shams at baseline (MD = 0.19, 95%CI: 0.03–0.34, *p* = 0.020), 4 h (MD = 0.95, 95%CI: 0.64–1.26, *p* < 0.001), 24 h (MD = 0.76, 95%CI: 0.43–1.09, *p* < 0.001), and 5d (MD = 0.34, 95%CI: 0.06–0.63, *p* = 0.020) post-injury. There was no effect of genotype or sex, nor any interactions on hindlimb slips.

### Elevated plus maze

**Fig. 4:**
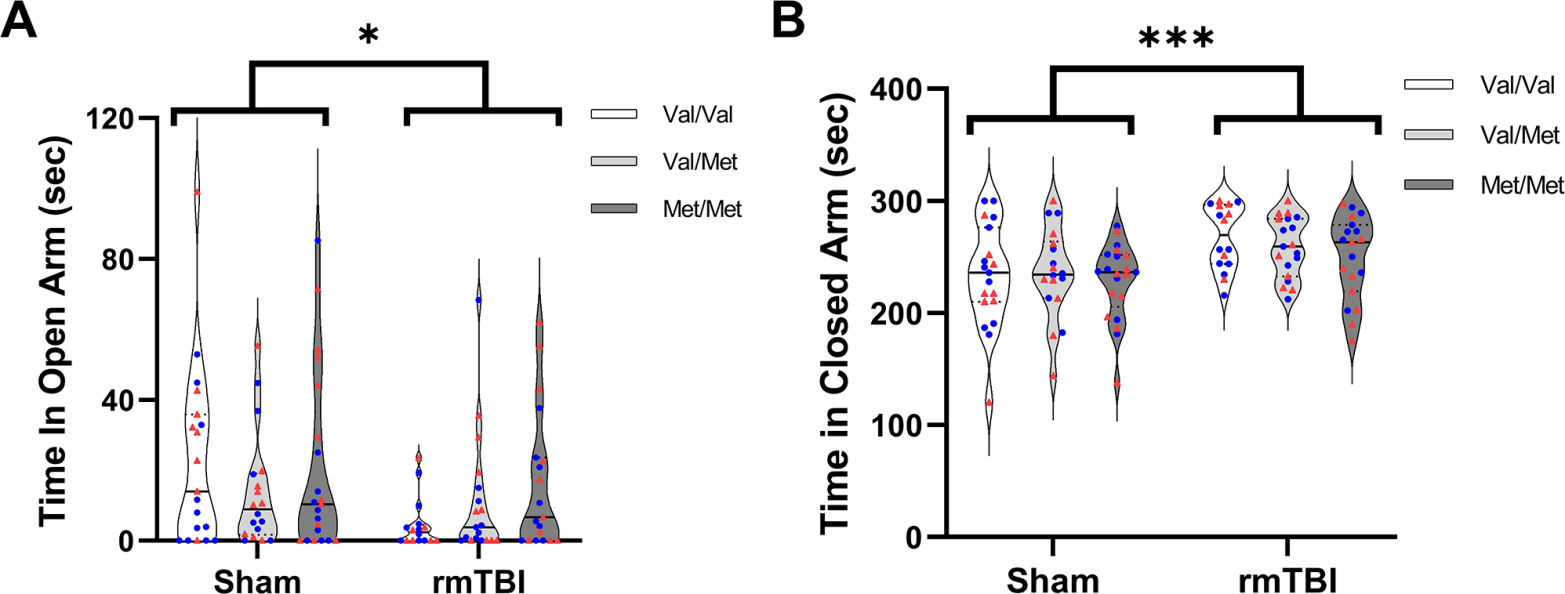
Anxiety-like response measured by the elevated plus maze. (**A**) Time spent in the open arms. Injured rats spent less time in the open arms compared with uninjured shams (*p* = 0.020). (**B**) Time spent in the closed arms. Injured rats spent more time in the closed arms compared with uninjured shams (*p* = 0.0002).  denotes males,  denotes females. Sham = 58 (Val/Val = 19, Val/Met = 18, Met/Met = 21), rmTBI = 54 (Val/Val = 16, Val/Met = 19, Met/Met = 19). Data presented as median/IQR. * indicates an effect of injury, *p* < 0.05, *** indicates an effect of injury, *p* < 0.001

As shown in Fig. 4, there was an effect of injury on time spent in the open arms, F(1,100) = 5.588, *p* = 0.020, as well as time spent in the closed arms, F(1,100) = 15.45, *p* = 0.0002, at 24 h. Injured animals spent more time in the closed arms and less time in the open arms compared with sham animals. There no effect of genotype or sex, nor any interaction on this task.

### Water maze

**Fig. 5:**
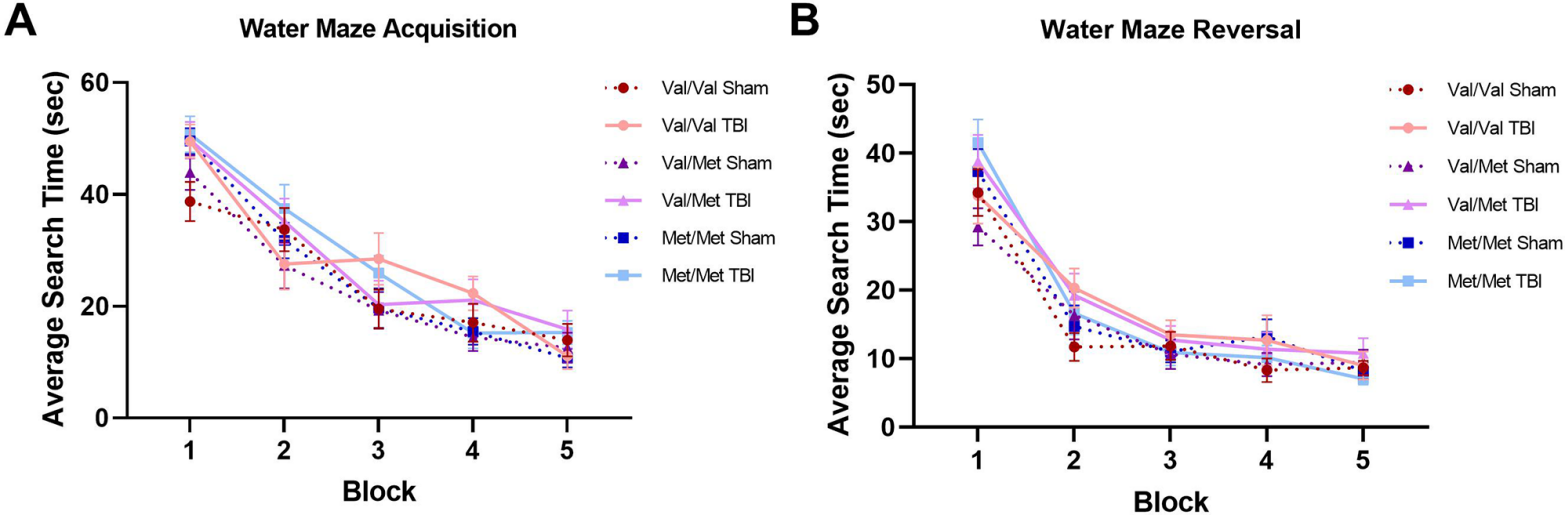
Spatial learning and memory as measured by water maze. (**A**) Average search time in the acquisition stage. There were no group differences in average search time. (**B**) Average search time in the reversal stage. There were no injury or genotype effects on reversal search time. Sham = 58 (Val/Val = 19, Val/Met = 18, Met/Met = 21), rmTBI = 54 (Val/Val = 16, Val/Met = 19, Met/Met = 19). Data presented as mean/SEM

The average search time in the acquisition and reversal stages of the water maze task are shown in Fig. 5A and B, respectively. For the search times in the acquisition stages and the reversal stages, Machley’s test of sphericity was violated (*p* < 0.001) and lower-bound corrections were used. Repeated measures ANOVAs demonstrated no effects of injury, genotype, or sex, nor interactions between variables for either of the search times for the acquisition or reversal stages of the water maze task.

### Hot/cold plate

**Fig. 6:**
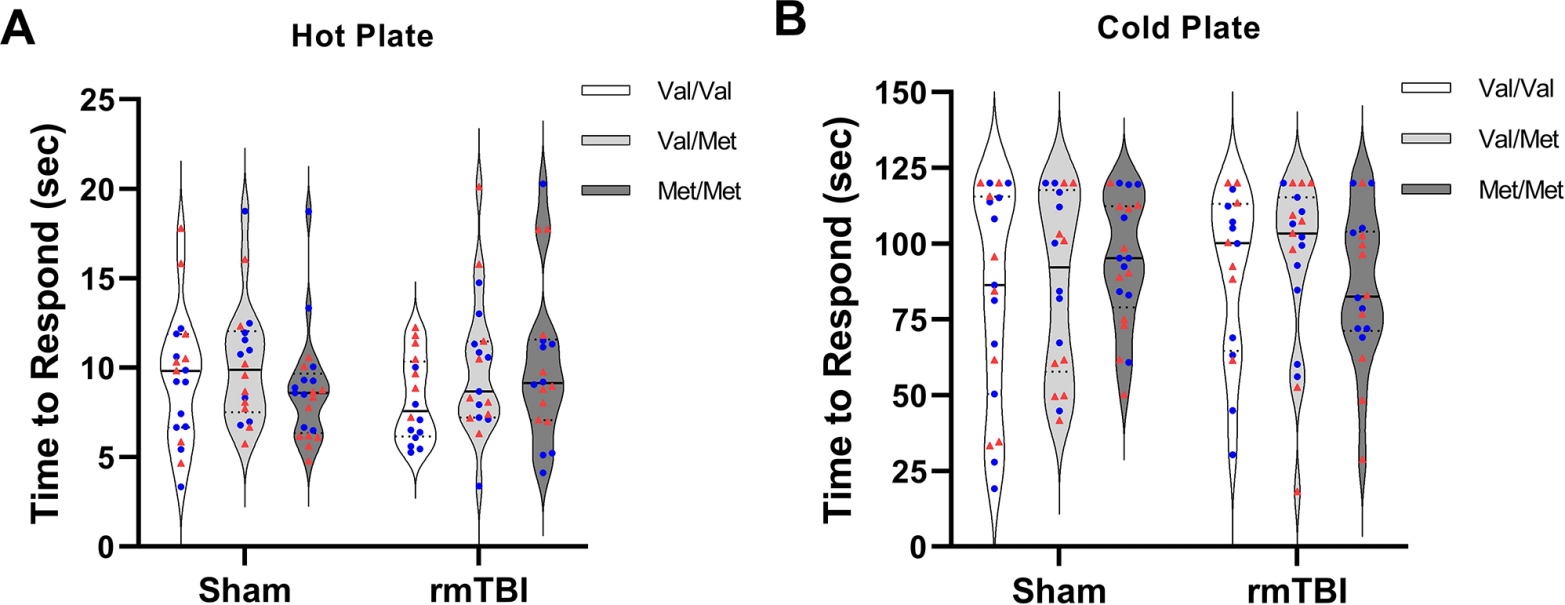
Nociceptive response measured by hot and cold plate. (**A**) Time to respond on hot plate. There were no group differences in time to respond on plate. (**B**) Time to respond on cold plate. There were no group differences in time to respond on cold plate.  denotes males,  denotes females. Sham = 58 (Val/Val = 19, Val/Met = 18, Met/Met = 21), rmTBI = 54 (Val/Val = 16, Val/Met = 19, Met/Met = 19). Data presented as median/IQR

Time to respond on a hot and cold plate is displayed in Fig. 6A and B, respectively. There was no effect of injury, genotype, sex nor an interaction between the three variables on time to response on either hot plate or cold plate.

### Plasma NfL

**Fig. 7:**
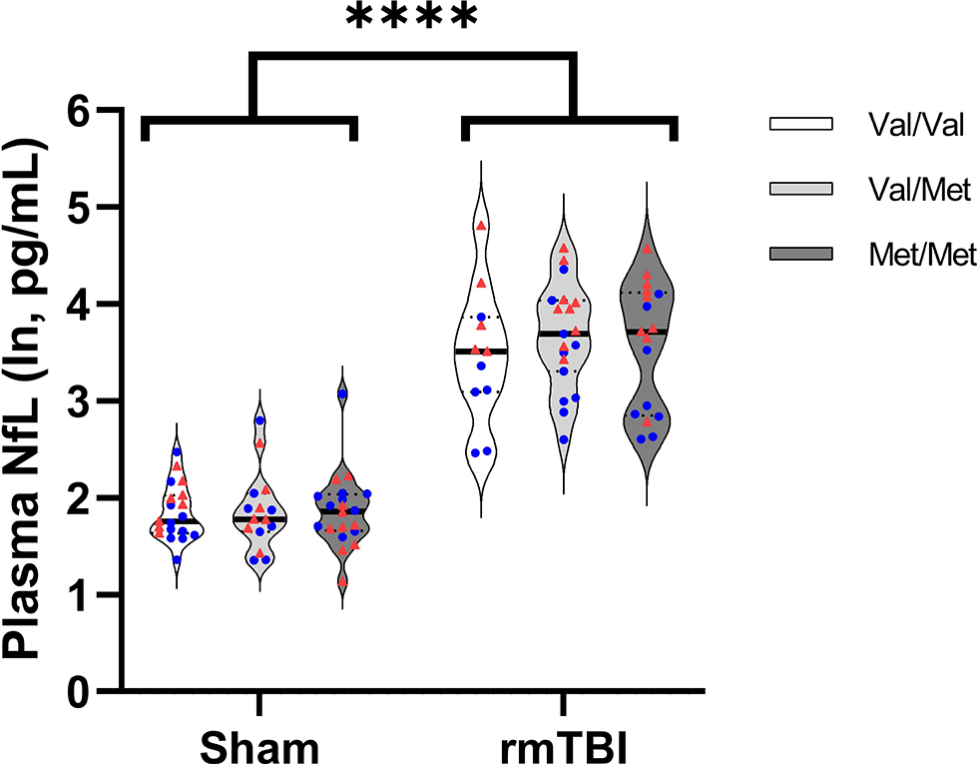
Plasma NfL levels at 10 days post-injury. Plasma NfL was elevated in injured rats at 10 days post-injury compared with shams.  denotes males,  denotes females. Sham = 58 (Val/Val = 19, Val/Met = 18, Met/Met = 21), rmTBI = 54 (Val/Val = 16, Val/Met = 19, Met/Met = 19). Data presented as median/IQR. **** indicates an effect of injury, *p* < 0.0001

There was an interaction between injury group and sex, F(1,89) = 17.19, *p* < 0.0001 with injured female rats having higher NfL levels than injured males (MD=-0.700, 95%CI:-0.987—0.413, *p* < 0.0001) (Fig. 7). There was no effect of genotype between groups.

### Iba-1 expression

**Fig. 8:**
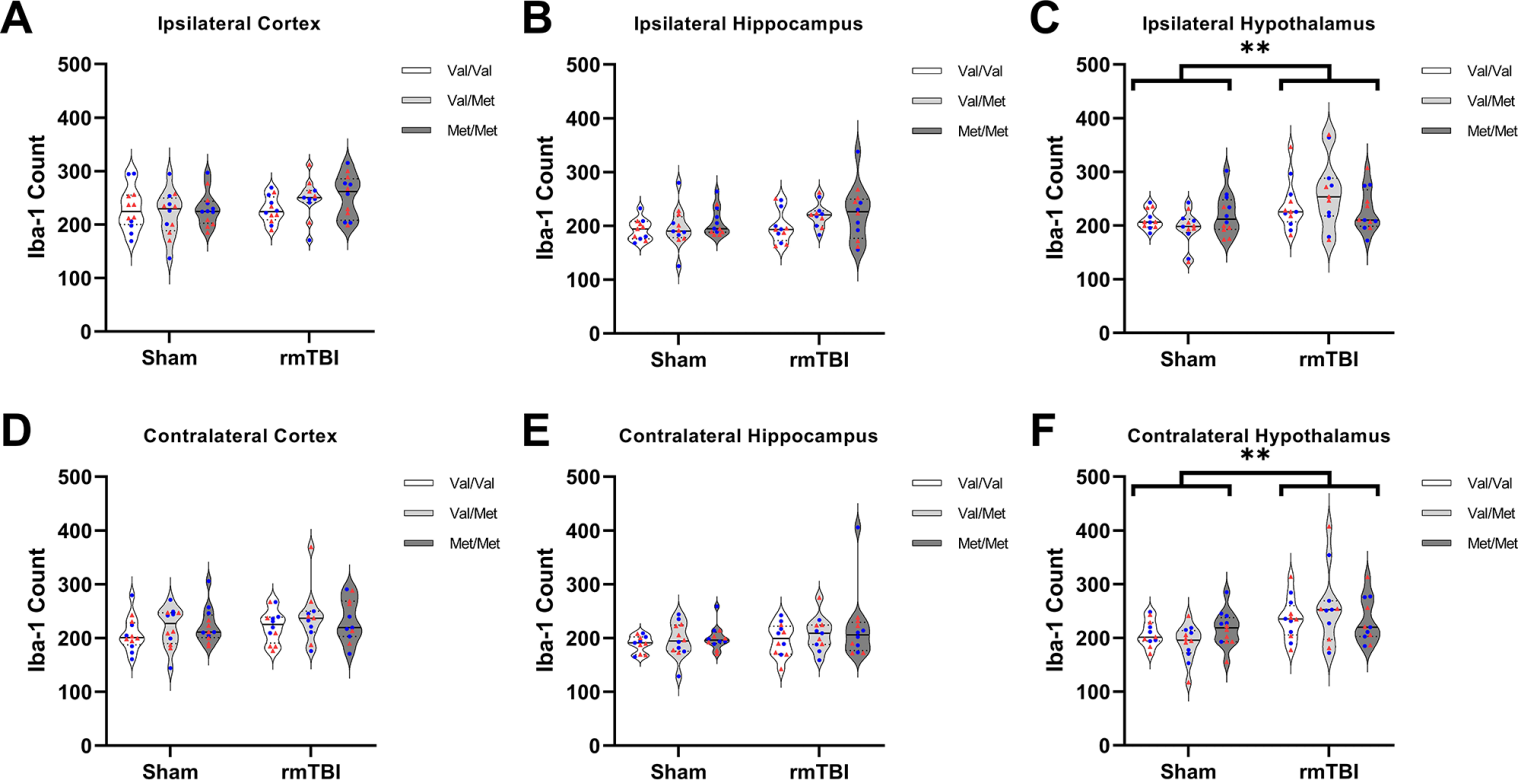
Microglial reactivity at 10 days post-injury. There was no effect of rmTBI on Iba-1 expression in the (**A**) ipsilateral cortex, (**B**) ipsilateral hippocampus, (**D**) contralateral cortex, or (**E**) contralateral hippocampus. Iba-1 labelling was increased in the (**C**) ipsilateral hypothalamus of rmTBI rats compared with shams (*p* = 0.0020). Similarly, Iba-1 labelling was increased in the (**F**) contralateral hypothalamus of rmTBI rats compared with shams (*p* = 0.0012).  denotes males,  denotes females. Sham = 36 (Val/Val = 12, Val/Met = 12, Met/Met = 12), rmTBI = 36 (Val/Val = 12, Val/Met = 12, Met/Met = 12). Data presented as median/IQR. ** indicates an effect of injury, *p* < 0.01

**Fig. 9:**
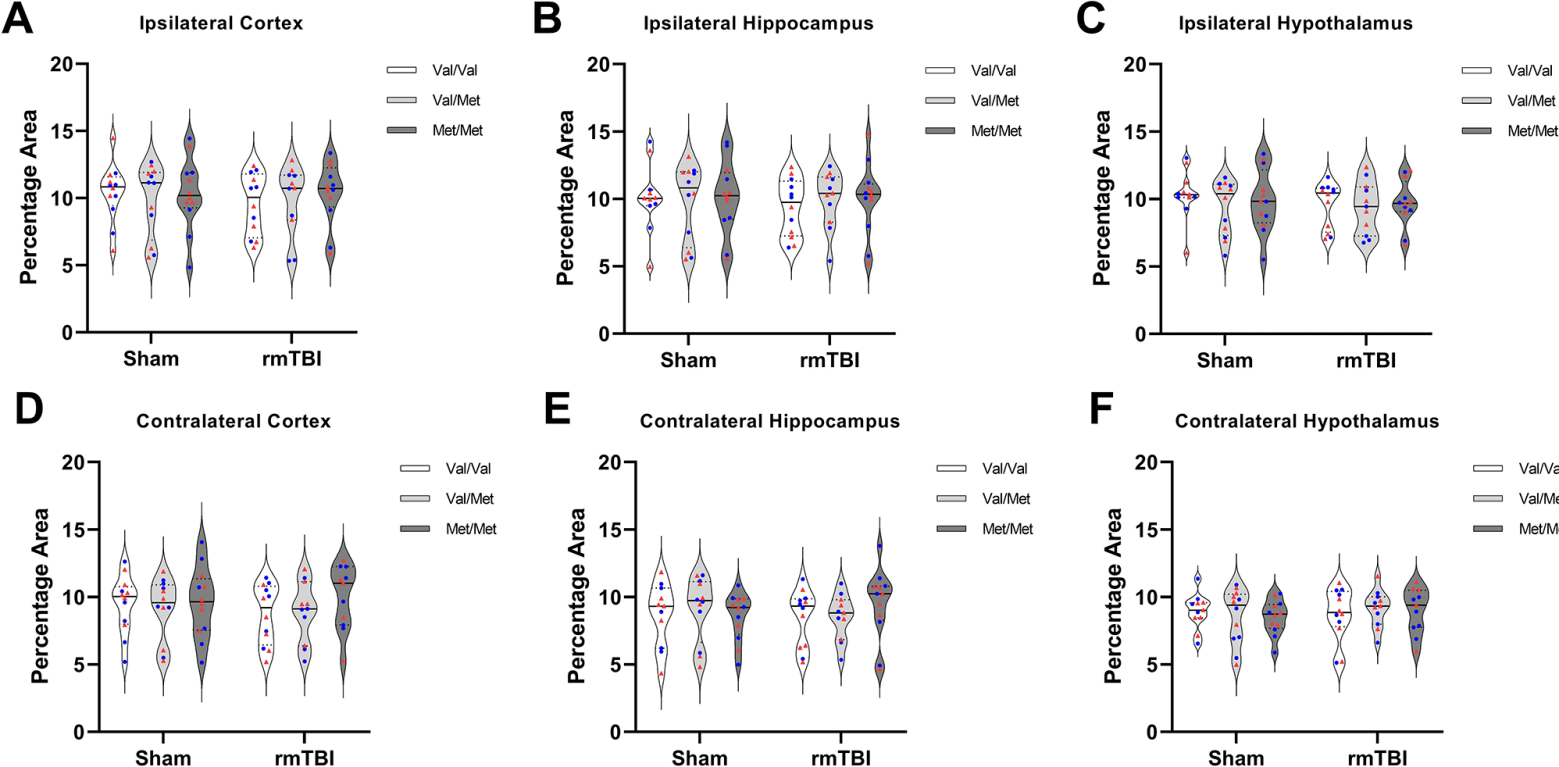
Percentage area of Iba-1 immunostaining. There was no effect of injury, genotype, nor sex on percentage area of Iba-1 expression in the (**A**) ipsilateral cortex, (**B**) ipsilateral hippocampus, (**C**) ipsilateral hypothalamus, (**D**) contralateral cortex, (**E**) contralateral hippocampus, or (**F**) contralateral hypothalamus.  denotes males,  denotes females. Sham = 36 (Val/Val = 12, Val/Met = 12, Met/Met = 12), rmTBI = 36 (Val/Val = 12, Val/Met = 12, Met/Met = 12). Data presented as median/IQR

The number of Iba-1 positive cells was higher in both the ipsilateral hypothalamus, F(1,58) = 10.49, *p* = 0.0020, and contralateral hypothalamus, F(1,57) = 11.68, *p* = 0.0012, of rmTBI rats compared with sham rats (Fig. 8). However, there was no effect of genotype or sex between groups, nor an interaction of the two in either region. There was no effect of injury, genotype, or sex, no any interaction of the variables on the number of Iba-1 positive cells in either the ipsilateral or contralateral cortex or hippocampus. No group differences or interactions were found in percentage area above the threshold for Iba-1 staining in any region analysed (Fig. 9).

## Discussion

The neurobehavioral and pathological consequences of rmTBI exposure remain poorly understood. However, research into the genetic contributions of rmTBI outcomes may serve to bridge gaps in our understanding of the underlying mechanisms causing neurological dysfunction. Our findings suggest that repetitive mTBI was acutely associated with deficits in sensorimotor function, as well as an increased anxiety-like response, and increased markers of axonal damage and neuroinflammation. However, no differences between Val68Met genotypes were observed in any of the behavioral measures, plasma NfL levels, nor Iba-1 expression. These results suggest the rs6265 polymorphism, while implicated in various neurological and neuropsychiatric conditions [43–46], may not exert a significant modulatory effect on the acute/subacute responses to rmTBI.

### Behavioral outcomes

Our results revealed substantial sensorimotor deficits in rats subjected to rmTBI, irrespective of their BDNF genotype, as evidenced by increased traverse times and hindlimb slips on the beam task. These deficits persist across multiple acute post-injury timepoints. While these results support previous findings that rmTBI results in acute sensorimotor impairments [47, 48], they stand in contrast to rodent BDNF studies that have shown Met carriers to have greater sensorimotor dysfunction compared with wildtype Val/Val carriers [27, 49]. These discrepancies may stem from differences in experimental design, including the injury model. Qin et al. (2014) found significant differences in motor function between genotypes in a chronic stroke model, where limb deficits are often a hallmark of the condition and potentially more likely to be evident than in mTBI [49, 50]. Anxiety-like behavior, as assessed by the elevated plus maze, also exhibited significant increases in injured rats, emphasising the potential influence of rmTBI on emotional response found in previous research [51]. BDNF Val68Met genotype was shown not to influence anxiety-like behaviors, which is consistent with the current animal literature [37, 39]. However, in human populations, the Val66Met genotype has been strongly associated with increased anxiety responses [18]. Therefore, it is important to consider that current behavioral tests used in animal models may not adequately capture nuanced emotional responses, such as anxiety. These tests often rely on broad measures of activity and exploration, which may fail to reflect the subtle variations in emotional states [52]. As a result, the complexity of anxiety-like behaviors, influenced by factors like injury severity and genotype, may be underestimated or overlooked in preclinical mTBI research. In addition, there were no cognitive impairments were observed in rats regardless of injury group or genotype. These results do not support those found by Giarratana et al. (2019) in which rmTBI resulted in worsened learning and memory in injured Met carrier mice relative to injured Val/Val mice [27]. However, these differences may be due to differences in protocol. While Giarratana et al. (2019) employed the standard 6-day Morris Water Maze protocol, we opted to utilise an abbreviated 2-day version that has been shown to reveal learning deficits in various TBI models [53–55]. The shortened protocol, while advantageous for reducing testing duration, may be less sensitive in detecting subtle cognitive impairments, potentially explaining the lack of significant differences observed in our study. The differences in results may also be due to the species evaluated. Previous research has shown that compared with mice, rats are superior in spatial learning and memory tasks due to their cognitive flexibility [56]. Therefore, it is possible that due to the more variable results seen in mice, genotype differences may have become more apparent in mice than in rats. Nociceptive response, as measured by the hot/cold plate, did not show significant alterations, suggesting that injury and genotype do not interact in attribution to altered pain perception.

### NfL

Plasma NfL levels, a marker of axonal injury, were significantly elevated in the rmTBI group, indicating the presence of neuroaxonal damage following repetitive injury. These results are consistent with previous work that has shown serum NfL elevations following rmTBI in rats [34]. The Val68Met genotype did not exert a significant influence on NfL levels, suggesting that the genetic marker may not contribute to the observed axonal injury in this study. However, previous research has shown NfL levels to peak at more acute timepoints than the 10d post-injury timepoint investigated in this study, and therefore, evaluating the influence of genotype on NfL levels at an earlier timepoint may yield more robust results in terms of genotype [40]. Initially, the 10d post-injury timepoint was selected for this study to allow for the evaluation of both NfL and BDNF levels, providing a more comprehensive understanding of the neuroaxonal damage and potential genetic influences of the injury response. However, due to limited blood volume following NfL analysis, there was insufficient sample available to test BDNF levels as originally intended. Additionally, faulty ELISA plates prevented the analysis of BDNF levels at 6 h post-injury. Despite this limitation, the selection of the 10d timepoint is still relevant, though not ideal, as it still aligns with the subacute phase of injury where persistent secondary injury mechanisms are still pronounced [34] and is supported by previous research demonstrating sustained NfL elevations at this timepoint following mTBI [41]. However, future studies should aim to optimize sample collection to enable analysis of a larger panel of markers in order to provide a more complete picture of the molecular processes underlying rmTBI in the context of genotype. However, it is also important to note that biomarkers such as GFAP and UCHL-1, while well-validated in clinical populations [57], do not have established commercial assays for use in rodents.

Female rats with rmTBI had significantly higher plasma levels of NfL compared with their male counterparts. To our knowledge, this is the first preclinical study to find this sex difference in NfL levels post-injury. Since male and female rats had impacts of the same magnitude, this finding suggests an increased neuroaxonal vulnerability in female rats. Nonetheless, it is worth noting that we measured NfL levels beyond the peak (previously shown to be 1-3d post-mTBI in this model) [40]. Therefore, the sex difference in NfL levels at 10d may be attributed to variations in brain clearance rates of NfL or ongoing axonal injury due to secondary injury cascades. Regarding the later, it is possible that sex differences in inflammation and oxidative stress may contribute [58, 59]. It is also important to note that sample size was determined based on prior research conducted by our research group using the same or similar TBI models and outcomes [34, 60], rather than power calculations. It is possible that a larger sample size is required to fully explore these subgroup differences.

### Iba-1

Microglia play a crucial role in neuroinflammation post-injury. In this study, the absence of genotype-dependent differences in Iba-1 expression does not support the prevailing hypothesis regarding the involvement of BDNF signalling in microglial activation following rmTBI. This discrepancy highlights the complexity of the relationship between genetic and pathophysiological factors, warranting further investigation into the mechanisms underlying these processes. Understanding the timing of microglial response post-injury is crucial to the interpretation of a neuroinflammatory response [55, 61]. Previous studies in rats with TBI have shown that timepoints more acute than 10 days may feature more prominent microglial activation [26]. A study specifically investigating the BDNF rs6265 SNP in a rodent model of rmTBI found increased activated microglia in Met carrier mice at 1- and 21d post-injury [27]. However, Giarratana et al. (2019) utilised the lateral fluid percussion injury model, which involved craniotomy and likely a more severe injury, which may have yielded more a significant inflammatory response. The model also involved anaesthesia of the animals with isoflurane prior to injury. It is worth noting that isoflurane exposure has been shown to alter signalling of the BDNF receptor, tropomyosin-related kinase receptor type B (TrkB), which may contribute to the genotype differences observed in their study [62]. In the current study, by selecting the 10d post-injury timepoint, we acknowledge that microglial responses may be closer to the peak of activation. However, this subacute timepoint is of interest because persisting inflammation has been associated with persisting symptoms following mTBI [63, 64]. In translational research aiming to inform clinical interventions, selecting a subacute post-injury timepoint allows researchers to investigate a critical period for clinical management. Understanding the microglial response during this window can provide valuable insights into the pathophysiological and genetic mechanisms underlying rmTBI and guide the development of targeted therapeutic strategies aimed at modulating inflammation.

## Conclusions

This study investigated the role of the Val68Met genotype in influencing the acute effects of repeated mTBIs in rats. While the observed behavioral and neuropathological changes may exhibit only nuanced influences from rs6265 genotype, they provide a basis for further exploration into the genetic contributions to rmTBI. Importantly, our study does not support the current literature conducted in mice [27], highlighting that there may be inter-species differences influencing outcomes and the need for caution when interpreting the translatability of findings. In addition, our study leverages a closed-head injury model, which better mimics the biomechanical forces and pathology typical of mTBI in humans [65]. Subsequent investigations into BDNF in the context of preclinical rmTBI should extend experiments to chronic timepoints to determine the effect of the Val68Met SNP on long-term outcomes of mTBI. Furthermore, it is imperative for future research to assess circulating levels of overall BDNF, as well as both pro- and mature BDNF, in conjunction with genotype analysis. This will enable a comprehensive exploration into the potential decreased secretion of BDNF due to the Val68Met genetic mutation. As the study of rmTBI progresses, the integration of genetic information into prognostic models may enhance our ability to predict and manage the short- and long-term consequences of rmTBI.

## Electronic supplementary material

Below is the link to the electronic supplementary material.

Supplementary Material 1: Fig. 1. Group breakdown by injury, genotype, and sex. Initially, 114 animals were used for the study. Following injuries, two animals were excluded due to mortality. One-hundred twelve animals underwent behavioral testing. A subset of 72 brains were used for immunohistochemistry.

Supplementary Material 2: Fig. 2. Iba-1 immunohistochemistry. Example of sham and rmTBI tissue slices used in analysis.

## Data Availability

The data that supports the findings of this study are available from the corresponding author upon reasonable request.

## Abbreviations

ACHI: Awake closed-head injury
ANOVA: Analysis of variance
BDNF: Brain-derived neurotrophic factor
CV: Coefficient of variation
EPM: Elevated plus maze
HCP: Hot/cold plate
Iba-1: Ionized calcium binding adaptor molecule 1
Met: Methionine
mTBI: mild traumatic brain injury
NfL: Neurofilament light
PBS: Phosphate-buffered saline
PFA: Paraformaldehyde
rmTBI: repetitive mild traumatic brain injury
RT: Room temperature
SNP: Single nucleotide polymorphism
TrkB: Tropomyosin-related kinase receptor type B
Val: Valine
WM: Water maze
