# Supplementary material for: Investigating the role of the brain-derived neurotrophic factor Val66Met polymorphism in repetitive mild traumatic brain injury outcomes in rats
Source: Behav Brain Funct. 2025 Mar 5;21:5. doi: 10.1186/s12993-025-00270-5 (PMC11884142; doi:10.1186/s12993-025-00270-5)
Supplement: Supplementary file 1 — Supplementary Material 1: Fig. 1. Group breakdown by injury, genotype, and sex. Initially, 114 animals were used for the study. Following injuries, two animals were excluded due to mortality. One-hundred twelve animals underwent behavioral testing. A subset of 72 brains were used for immunohistochemistry. [file 12993_2025_270_MOESM1_ESM.docx]

**Immunohistochemistry**

**Sham Rats: 36**

|  | **Male** | **Female** |
| --- | --- | --- |
| **Val/Val** | 6 | 6 |
| **Val/Met** | 6 | 6 |
| **Met/Met** | 6 | 6 |

**rmTBI Rats: 36**

|  | **Male** | **Female** |
| --- | --- | --- |
| **Val/Val** | 6 | 6 |
| **Val/Met** | 6 | 6 |
| **Met/Met** | 6 | 6 |

**rmTBI Rats: 54**

|  | **Male** | **Female** |
| --- | --- | --- |
| **Val/Val** | 9 | 7 |
| **Val/Met** | 9 | 10 |
| **Met/Met** | 10 | 9 |

**Sham Rats: 58**

|  | **Male** | **Female** |
| --- | --- | --- |
| **Val/Val** | 11 | 8 |
| **Val/Met** | 9 | 9 |
| **Met/Met** | 10 | 11 |

**Behavioral Battery**

**2 Rats Excluded – Mortality**

|  | **Male** | **Female** |
| --- | --- | --- |
| **Met/Met** | **1** | **1** |

**Total Rats: 114**

|  | **Male** | **Female** |
| --- | --- | --- |
| **Val/Val** | 20 | 15 |
| **Val/Met** | 18 | 19 |
| **Met/Met** | 21 | 21 |

**Sham/rmTBI**
