# Supplementary figures and images for: Investigating the role of the brain-derived neurotrophic factor Val66Met polymorphism in repetitive mild traumatic brain injury outcomes in rats
Source: Behav Brain Funct. 2025 Mar 5;21:5. doi: 10.1186/s12993-025-00270-5 (PMC11884142; doi:10.1186/s12993-025-00270-5)

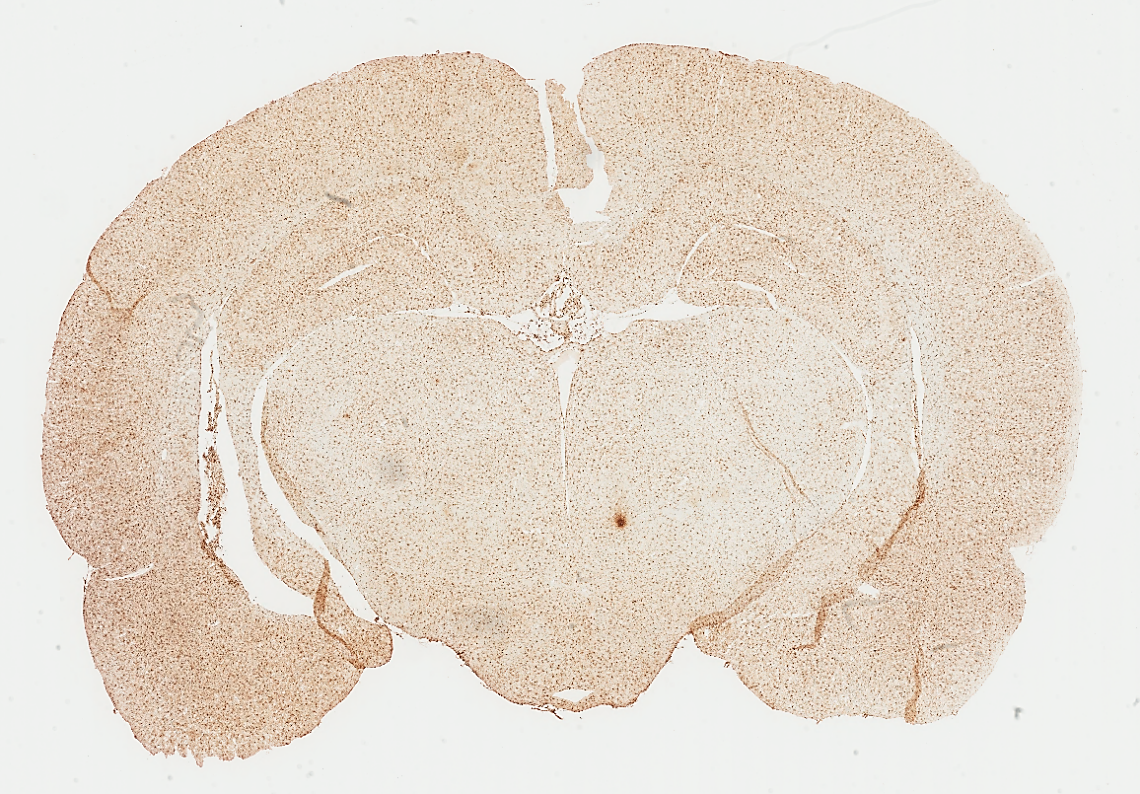


**Sham**


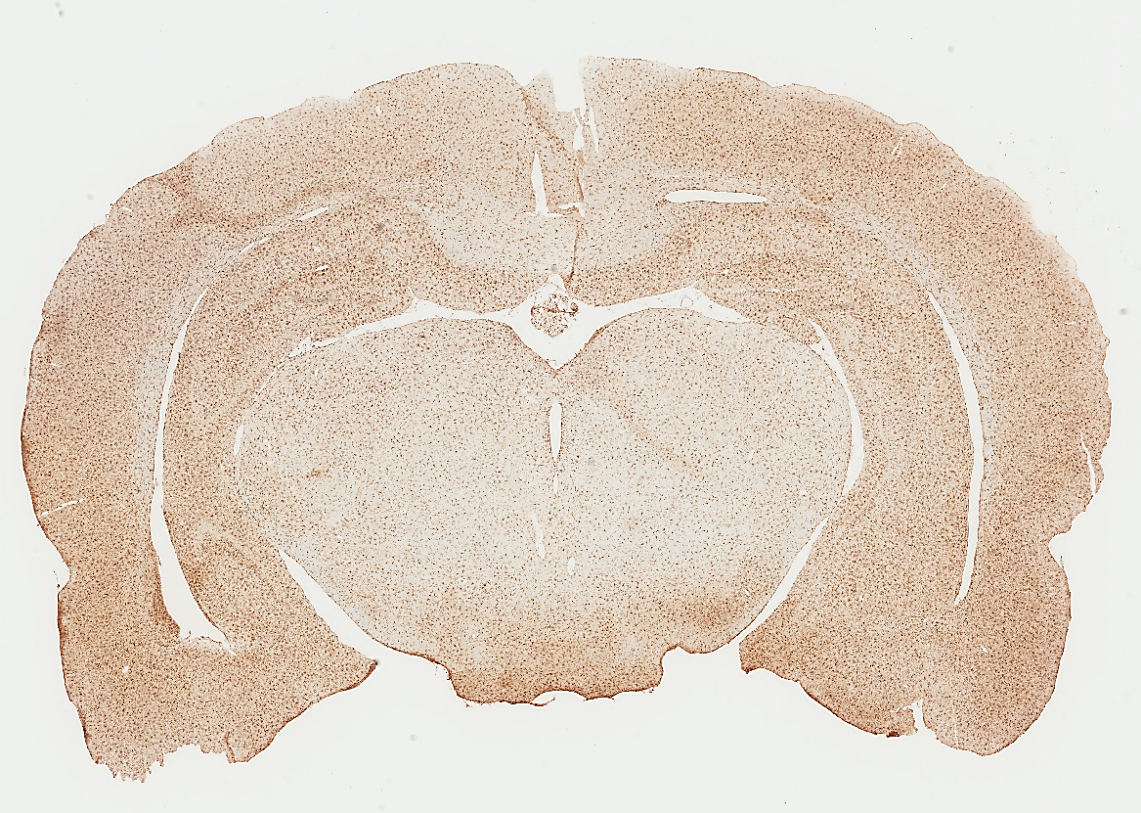


**rmTBI**

Supplement: Supplementary file 2 — Supplementary Material 2: Fig. 2. Iba-1 immunohistochemistry. Example of sham and rmTBI tissue slices used in analysis. [file 12993_2025_270_MOESM2_ESM.docx]
